# Effect of low skeletal muscle mass on long-term mortality after abdominal aortic aneurysm repair: A meta-analysis

**DOI:** 10.12669/pjms.39.2.7366

**Published:** 2023

**Authors:** Junjing Chen, Yanfen Xia, Yi Liu, Huifang Zhu

**Affiliations:** 1 Junjing Chen, Department of Thyroid Surgery and Vascular Surgery, Huzhou Central Hospital, Affiliated Central Hospital HuZhou University, Huzhou 313000, Zhejiang Province, P.R. China; 2 Yanfen Xia, Department of Thyroid Surgery and Vascular Surgery, Huzhou Central Hospital, Affiliated Central Hospital HuZhou University, Huzhou 313000, Zhejiang Province, P.R. China; 3 Yi Liu, Department of Thyroid Surgery and Vascular Surgery, Huzhou Central Hospital, Affiliated Central Hospital HuZhou University, Huzhou 313000, Zhejiang Province, P.R. China; 4 Huifang Zhu, Department of Thyroid Surgery and Vascular Surgery, Huzhou Central Hospital, Affiliated Central Hospital HuZhou University, Huzhou 313000, Zhejiang Province, P.R. China

**Keywords:** Sarcopenia, Psoas muscle, Aortic aneurysm, Mortality, Skeletal mass, Malnutrition, Vascular surgery

## Abstract

**Objective:**

This meta-analysis was designed to assess if pre-operative low skeletal muscle mass impacts mortality rates of patients undergoing abdominal aortic aneurysm (AAA) repair.

**Methods:**

Datasets of PubMed, CENTRAL, ScienceDirect, Embase, and Google Scholar were searched from 1^st^ January 1980 to 15^th^ December 2021 for studies assessing the role of low skeletal muscle mass on mortality rates of AAA repair. Studies measuring skeletal muscle mass on computed tomography scans and reporting long-term mortality (>1 year) were included. Multivariable adjusted ratios were combined in a random-effects model.

**Results:**

Fifteen studies with 3776 patients were included. Meta-analysis showed a statistically significant increased risk of all-cause mortality in patients with low skeletal muscle mass (HR: 2.07 95% CI: 1.56, 2.74 I^2^=65% p<0.00001) as compared to normal muscle mass patients. Pooled data indicated that low skeletal muscle mass was associated with statistically significant increased risk of mortality in studies on endovascular repair (HR: 2.86 95% CI: 1.95, 4.20 I^2^=58% p<0.00001) as well as those including a mixed group of patients (HR: 1.39 95% CI: 1.06, 1.82 I^2^=31% p=0.02).

**Conclusion:**

Low skeletal muscle mass in AAA patients undergoing surgical repair is associated with increased risk of long-term mortality. Current evidence is limited by the retrospective nature of data and variability in defining and measuring low skeletal muscle mass. There is a need for future prospective studies defining the optimal cut-off of low skeletal muscle mass in different populations.

## INTRODUCTION

Abdominal aortic aneurysms (AAA) are an important cause of mortality in the elderly and are usually associated with risk factors like family history, hypertension, tobacco use, and male gender.^1^ The majority of AAA are asymptomatic, being detected incidentally with ultrasound or computed tomographic (CT) scans. However, continuing subclinical growth may lead to symptoms like abdominal or back pain, thromboembolization, atheroembolization, arteriovenous or aortoenteric fistula, and even life-threatening aortic rupture.^2^ Elective surgical repair of AAA is usually recommended when the diameter of the aneurysm is >5.5cm.^1^,^3^ Indeed, as compared to open repair, endovascular repair of AAA has significantly reduced the morbidity and mortality associated with the procedure.^4^

However, since the majority of patients with AAA are aged and frail, vascular surgeons need to stratify patients based on the presence of comorbidities and other risk factors which may worsen postoperative outcomes.^5^ One such risk factor which has gained importance in recent times is frailty. Fraility is as an age-related syndrome characterized by unintended loss of weight, tiredness, weakness, slow gait, and reduced physical activity.^6^ Since the functional decline associated with frailty can be delineated by the morphological or quantitative change of sarcopenia, several researchers consider sarcopenia as an indicator of preoperative frailty.^7^ Indeed, sarcopenia is now a recognized condition characterized by a progressive and generalized loss of skeletal muscle mass and strength. Quantification of low skeletal muscle mass is easy and usually based on cross-sectional imaging of psoas muscles.^8^

Research has shown that reduced skeletal muscle mass is closely related to adverse outcomes in patients with a variety of diseases ranging from cardiac ailments to malignancies.^9^-^12^ Recently, several studies have attempted to explore the relationship between low skeletal muscle mass and AAA repair, albeit with conflicting results. While some studies^13^,^14^ have noted a strong correlation between low skeletal muscle mass and poor survival others^15^,^16^ have shown that low skeletal muscle mass cannot be used as a prognostic indicator in patients undergoing AAA repair. Because these individual studies were of a small sample size, a pooled analysis was conducted by Antoniou et al^17^ to improve the quality of evidence. However, their review could include only seven studies. With new literature^18^-^20^ published in recent years, there is a requirement for updated evidence. Therefore, the objective of this updated meta-analysis was to assess if low skeletal muscle mass is associated with long-term mortality in patients undergoing AAA repair.

## METHODS

The protocol of this meta-analysis was prospectively on the online database PROSPERO (CRD42022295885). The reporting guidelines of the PRISMA statement (Preferred Reporting Items for Systematic Reviews and Meta-analyses) were adhered.^21^

### Literature search:

Two reviewers electronically searched the datasets of PubMed, CENTRAL, Embase, ScienceDirect, and Google Scholar databases from 1^st^ January 1980 to 15^th^ December 2021. We utilized both free-text and MeSH keywords for the literature search, namely, “sarcopenia”, “psoas muscle”, “skeletal muscle” and “abdominal aortic aneurysm” in various combinations (Supplementary Table-I). After the search, we electronically deduplicated the results and screened articles using the titles and abstracts to identify appropriate studies. The identified articles were read completely by two reviewers for final inclusion. Any discrepancies in study selection were resolved by consensus.

**Supplementary Table-I:** Search strategy.

Query	Search Details
(abdominal aortic aneurysm) AND (skeletal muscle)	(“aortic aneurysm, abdominal"[MeSH Terms] OR (“aortic”[All Fields] AND “aneurysm”[All Fields] AND “abdominal”[All Fields]) OR “abdominal aortic aneurysm"[All Fields] OR (“abdominal”[All Fields] AND “aortic”[All Fields] AND “aneurysm”[All Fields])) AND (“muscle, skeletal"[MeSH Terms] OR (“muscle”[All Fields] AND “skeletal”[All Fields]) OR “skeletal muscle"[All Fields] OR (“skeletal”[All Fields] AND “muscle”[All Fields]))
(abdominal aortic aneurysm) AND (psoas muscle)	(“aortic aneurysm, abdominal"[MeSH Terms] OR (“aortic”[All Fields] AND “aneurysm”[All Fields] AND “abdominal”[All Fields]) OR “abdominal aortic aneurysm"[All Fields] OR (“abdominal”[All Fields] AND “aortic”[All Fields] AND “aneurysm”[All Fields])) AND (“psoas muscles"[MeSH Terms] OR (“psoas”[All Fields] AND “muscles”[All Fields]) OR “psoas muscles"[All Fields] OR (“psoas”[All Fields] AND “muscle”[All Fields]) OR “psoas muscle"[All Fields])
(abdominal aortic aneurysm) AND (sarcopenia)	(“aortic aneurysm, abdominal"[MeSH Terms] OR (“aortic”[All Fields] AND “aneurysm”[All Fields] AND “abdominal”[All Fields]) OR “abdominal aortic aneurysm"[All Fields] OR (“abdominal”[All Fields] AND “aortic”[All Fields] AND “aneurysm”[All Fields])) AND (“sarcopenia”[MeSH Terms] OR “sarcopenia”[All Fields])

### Inclusion criteria:

- All types of studies conducted on patients undergoing AAA repair (open or endovascular).
- Studies were to assess the role of low skeletal muscle mass on survival of AAA patients.
- Skeletal muscle mass was to be measured on CT scans that were no older than 12 months from AAA repair. Psoas and adjacent muscles measured at the level of lumbar vertebrae were acceptable for inclusion.
- Studies were to define low skeletal muscle mass and compare mortality between low vs non-low muscle mass. No restriction was placed on this criterion and all definitions of low skeletal muscle mass by the included studies were acceptable.
- Mortality data was reported as adjusted ratio with 95% confidence intervals (CI).
- Duration of the follow-up was >1 year.

### Exclusion criteria:

- Studies on mixed AAA and thoracoabdominal aneurysm patients.
- Studies not using CT to measure low skeletal muscle mass.
- Studies not reporting adjusted data.
- Non-English language studies.
- Studies combining skeletal muscle mass and attenuation.

### Data extraction:

The following data was noted: author details, type of study and its database, sample size, patients with low skeletal muscle mass, demographic details, type of AAA repair (open or endovascular), the cut-off for defining low skeletal muscle mass, method and level of measurement, and follow-up.

The included studies either defined low skeletal muscle based on a cut-off value derived from receiver operating curve (ROC) analysis or from the literature, which was then used to group low and normal skeletal muscle mass, or the study authors classified skeletal muscle mass into different tertiles (low, medium, high) in which case the lowest tertile was considered to be low skeletal muscle mass for our meta-analysis. This methodology for data analysis is similar to the prior published meta-analysis.

### Quality assessment:

The Newcastle-Ottawa scale (NOS)^22^ was used by two reviewers to examine the studies on the selection of study population, comparability, and outcomes. Stars were given for each domain with a maximum score of nine. Nine points indicated low risk of bias, seven to eight points indicated moderate while <6 indicated high risk of bias.

### Statistical analysis:

The meta-analysis was performed using “Review Manager” (RevMan, version 5.3; Nordic Cochrane Centre [Cochrane Collaboration], Copenhagen, Denmark; 2014). Adjusted ratios of mortality was pooled to calculate hazard ratios (HR) with 95% CI using the random-effects model. Heterogeneity was judged by the I^2^ statistic and publication bias by inspection of funnel plots. A sensitivity analysis was also conducted. Subgroup analyses was done based on the type of AAA repair.

## RESULTS

**Fig.1:**
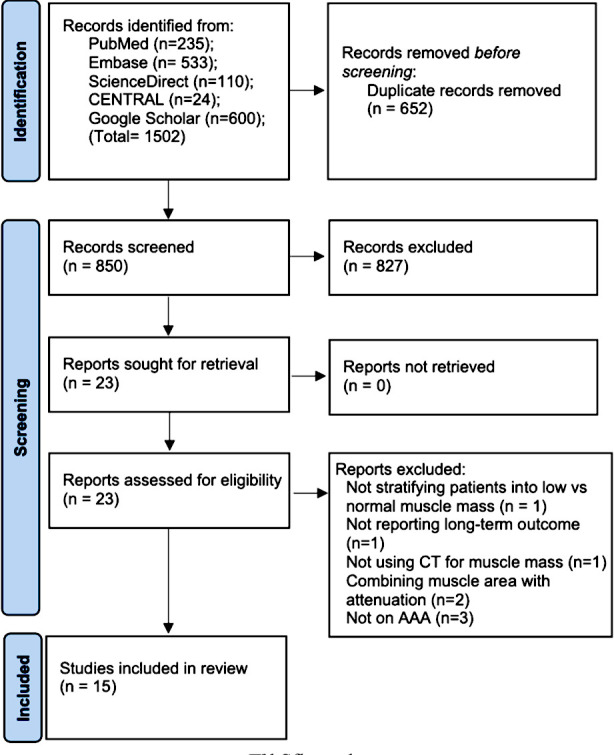
Study flow-chart

One thousand five hundred two (1502) articles were searched in total (Fig.1), in which 23 articles were chosen for full-text analysis. Eight articles were excluded and the remaining fifteen studies were found to be eligible for this meta-analysis.^13-16,18-20,23-30^ The majority of the studies were retrospective cohort in nature (Table-I). A total of 3776 patients were included in the studies with the sample size ranging from 103 to 489 patients. One study included patients undergoing only open AAA repair while nine studies included patients undergoing only endovascular repair.

**Table-I:** Details of included studies

Study	Database	Study type	Study period	Sample size	Patients with low skeletal mass	Mean/ Median age (years)	Male gender (%)	EVAR (%)	Muscle area cut-off for low skeletal mass	Level of psoas muscle area measurement	Method of measurement	Follow-up	NOS score
Lee 2011^27^	University of Michigan, USA	NR	2000-2008	262	84	69.5	76.7	0	NR	Superior aspect of L4	Software based	2.3 years	8
Drudi 2016^26^	Jewish General Hospital, Canada	R	2010-2015	149	49	75.6	84	85	21.7 cm^2^for men; 13.5 cm^2^for women	Top of L4	Software based	22.4 months ± NR	8
Hale 2016^13^	Greenville Health System, USA	R	1999-2007	200	25	74	87.5	100	114 cm^2^for men; 89.8 cm^2^for women (inclusive of abdominal wall, spinal and psoas muscle)	Central level of L3	Manual segmentation of muscle groups	Median 8.4 years [IQR 5.3-11.7]	8
Indrakusuma 2018^16^	Academic Medical Centre, Netherlands	R	2007-2013	124	31	69	87.1	62	14.6cm^2^	Central level of L3	NR	Up to 5 years	8
Newton 2018^25^	Veterans Affairs Medical Left, USA	NR	2010-2016	134	45	70	100	100	240.6cm^2^	Immediately inferior to L4 superior endplate	Manual outline tool	Median 27 months [IQR 18-40]	8
Thurston 2018^14^	Multicentric, Australia	R	2008-2013	191	30	NR	NR	100	50cm^2^/ m^2^	Most caudal aspect of L3	Manual tracing	Up to 5 years	8
Huber 2019^24^	University of Virginia, USA	R	2010-2017	407	NR	72.2	84.3	100	14.2cm^2^	Superior aspect of L4	Manual tracing	39± 33.5 months	8
Waduud 2019^15^	National Vascular Registry, UK	P	2010-2016	380	110	75	87.2	66.2	5.5cm^2^/ m^2^for men; 45cm^2^/ m^2^for women	L3	NR	2.7± 2.7 years	8
Smoor 2020^19^	St Antonius Hospital, Netherlands	R	2012-2018	489	NR	71.6	86.5	63.8	45.1cm^2^/ m^2^for men; 37.8cm^2^/ m^2^for women	L3	Manual tracing	Median 42 months [IQR 24-59.9]	8
Alenezi 2021^18^	Toronto General Hospital, Canada	R	2008-2019	257	86	75.4	75.1	100	17.4 cm^2^for men; 10.6 cm^2^for women	Mid-level of L3	Manual tracing	32.7 months ± NR	8
Bang 2021^23^	Asan Medical Left, Korea	R	1999-2011	379	104	69	89.4	44.1	39.6cm^2^/ m^2^for men; 28.6cm^2^/ m^2^for women	Most caudal aspect of L3	Software based	Median 3.3 years [IQR 1.6-5.2]	8
Cheng 2021^30^	Northwestern University Feinberg School of Medicine, USA	R	2002-2014	272	50	72	87.1	100	5.3cm^2^/ m^2^	Mid-level of L3	Software based	Up to 5 years	8
Ikeda 2021^20^	Nagoya University Hospital, Japan	R	2007-2013	324	166	78	85	100	16cm^2^	L4	Software based	Median 56.7 months	8
Ito 2021^28^	The Jikei University Kashiwa Hospital, Japan	R	2011-2018	103	NR	76	84	100	48.2cm^2^/ m^2^for men; 36.8cm^2^/ m^2^for women	Most caudal aspect of L3	Software based	Median 35.3 months	8
Oliveira 2021^29^	Centro Hospitalar e Universitario de Coimbra, Portugal	R	2014-2018	105	35	72.9	100	100	12.07cm^2^	L3	Software based	27.6± 15.6 months	8

P, prospective; R, retrospective; NR, not reported; L, lumbar vertebrae; EVAR, endovascular aneurysm repair.

There was wide variation in the studies in defining low skeletal muscle mass. All studies measured defined skeletal muscle area by measuring the bilateral psoas muscles with or without additional muscular structures. Measurements were made on CT at the level of L3 or L4 vertebrae using manual or software-based tracing tools. The follow-up duration was also variable amongst the included studies. The NOS score of the included studies was eight indicating a moderate risk of bias.

### Meta-analysis:

**Fig.2:**
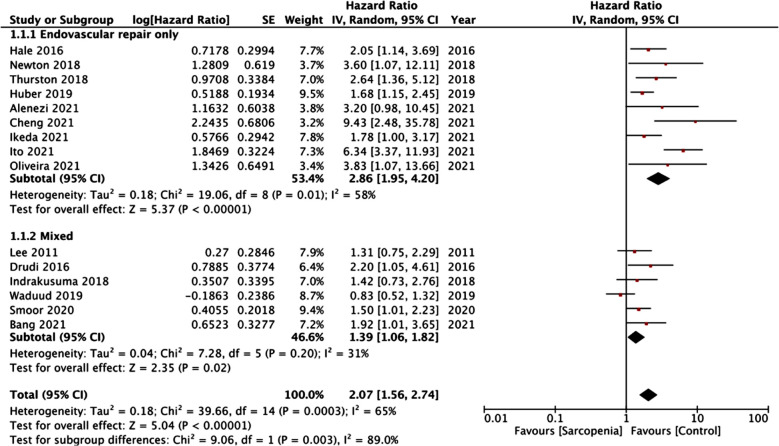
Meta-analysis of the impact of low skeletal muscle mass on long-term mortality after AAA repair with subgroup analysis based on the type of repair.

**Fig.3:**
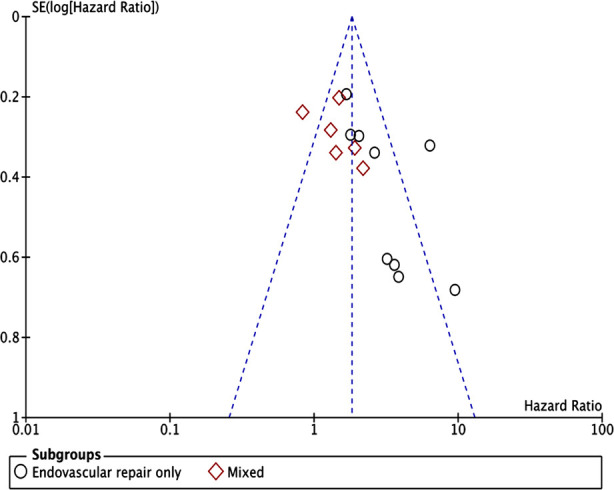
Funnel plot for the meta-analysis of low skeletal muscle mass and mortality after AAA repair

On pooled analysis of all 15 studies, we noted a statistically significant increased risk of all-cause mortality in patients with low skeletal muscle mass (HR: 2.07 95% CI: 1.56, 2.74 I^2^=65% p<0.00001) as compared to normal muscle mass patients (Fig.2). The results did not deviate on sensitivity analysis. There was no evidence of publication bias on visual inspection of the funnel plot (Fig.3). Based on the type of AAA repair, we segregated the included studies into two groups: Endovascular repair only and a mixed group (including both open and endovascular repair).

On subgroup analysis, a significant effect of low skeletal muscle mass was noted in both groups. Pooled data indicated that low skeletal muscle mass was associated with statistically significant increased risk of mortality in studies on endovascular repair (HR: 2.86 95% CI: 1.95, 4.20 I^2^=58% p<0.00001) as well as those including a mixed group of patients (HR: 1.39 95% CI: 1.06, 1.82 I^2^=31% p=0.02) (Fig.2).

## DISCUSSION

In line with the growing trend of minimally invasive surgical procedures, the rate of endovascular repair has risen sharply from 5.2% to 74% over the last decade with a corresponding decline in mortality rates.^31^ Compared to open surgery, early survival has significantly improved with endovascular repair, however, its impact on long-term outcomes is still questionable. Antoniou et al^32^ in a meta-analysis have demonstrated that endovascular repair is associated with a significantly higher risk of mortality as compared to open repair on long-term follow-up. Also, long-term survival after AAA repair is not solely dependent on the type of repair but many other modifiable risk factors.^33^

Accurate identification of such patient-specific prognostic factors would aid clinicians in patient counseling, risk stratification, and providing a patient-specific treatment plan. In this context, sarcopenia, as a surrogate marker of frailty, has gained widespread research interest as it can be easily calculated based on the cross-sectional area of the psoas muscle which in turn is well correlated with whole-body muscle mass. A meta-analysis by Zhang et al^12^ has shown that sarcopenia was significantly associated with increased risk of mortality in critically ill patients in the intensive care unit. Another review by Xue et al^11^ has demonstrated an increased risk of major adverse cardiac events in patients with coronary artery disease and sarcopenia. Similarly, Weerink et al^9^ have shown that low skeletal muscle mass increases risk of postoperative complications in cancer patients.

Similarly, our meta-analysis also noted that low skeletal muscle mass is associated with poor survival in patients undergoing AAA repair. A pooled analysis of data from 3776 patients indicated a statistically significant increased risk of long-term mortality with low skeletal muscle mass. The overall pooled effect size, inclusive of all types of studies, was 2.07 indicating a two times increased risk of mortality. The strength of the evidence is gauged by the fact that no study was found to have an undue influence on the pooled results with no change in the significance of the effect size on sensitivity analysis.

On examination of the forest plot, it can be noted that while a few studies indicated no impact of low skeletal muscle mass on mortality rates the direction of the results was more or less consistent across the included studies. We also noted that the risk of mortality was slightly on the higher side when only endovascular studies were pooled together (HR:2.86). In contrast in the subgroup of mixed studies (open and endovascular), the overall effect size was just 1.39 which, however, still indicated poor survival amongst low skeletal muscle mass patients. This difference in the two groups is difficult to explain since there was just one study that included only open repair patients and it is unclear if the prognostic role of low skeletal muscle mass is altered based on the type of repair.

The results of our study concur with the previous review of Antoniou et al^17^ which too have demonstrated poor survival in low skeletal muscle mass patients undergoing AAA repair. However, by adding eight new studies, the current meta-analysis presents significantly updated evidence. Our results are further supported by studies not included in the meta-analysis. Kays et al^34^ in a cohort of 505 patients undergoing AAA repair have demonstrated a significantly increased risk of early mortality with sarcopenia. Kärkkäinen et al^35^ have suggested that in addition to muscle area, muscle quality is an important predictor of prognosis. In their two recently published studies,^35^,^36^ the authors combined psoas muscle cross-sectional area with radio density and demonstrated that low muscle mass and density were associated with an increased risk of mortality and complications.

A major limitation in the interpretation and clinical application of our results is the variability of definition and technique to measure low skeletal muscle mass. The included studies divided their samples either into two groups based on a singular cut-off value or into different tertiles with the lowest tertile being considered as low skeletal muscle mass. The cut-off value was widely different across studies with some studies adjusting it for gender and height while others did not. The level of measurement, the area of measurement, and the technique of measurement (manual or software-based) also differed across studies. Defining sarcopenia has indeed been a challenge since the recognition of this disease.^37^

The most recent European consensus on definition and diagnosis of sarcopenia (EWGSOP2) has a three-step, cut-off defined structure for diagnosing sarcopenia: first, screening by the SARC-F questionnaire; second, diagnosis by low muscle mass and strength; and third, severity grading by physical performance.^38^ However, EWGSOP2 has recognized that the cut-off points for low muscle mass and strength still depend upon the measurement techniques and availability of studies, and disagreements over cut-off points have hindered research and development in the field.

They have recommended using normative data from the study population till future research identifies specific validated cut-off points in the global population.^38^ It is therefore important to recognize that since the cut-off value of each study was different and dependent on their study population, it is not possible to utilize the results of the current meta-analysis into clinical practice and convert the evidence into a morphometric stratification system. The current data only provides a guide to clinicians that low skeletal muscle mass is a poor prognostic factor in AAA patients but the definition of low skeletal muscle mass has to be derived and validated by clinicians in their respective patient populations.

### Limitations:

Firstly, the majority data in the meta-analysis was retrospective in nature. Retrospective studies are prone to selection bias, errors in record keeping, and data entry. Secondly, our meta-analysis could only assess the impact of low skeletal muscle mass on mortality. Lack of data on other important outcomes like complications precluded a meta-analysis. Thirdly, long-term patient survival after AAA repair depends on several confounding factors. While our meta-analysis only pooled adjusted data, it is plausible that some known and unknown confounders could have been missed by the included studies and skewed the study results.

### Strength:

Nevertheless, the strength of the meta-analysis lies in the large number of studies included in the analysis. By including several recent studies, our study presents the most comprehensive and updated evidence on the topic. Our findings have important implications for clinical practice. Since CT scans are routinely ordered for AAA patients, psoas muscle cross-sectional area can be easily measured in all patients undergoing surgical intervention. Such data would help in risk stratification and taking informed clinical decisions.

## CONCLUSION

Results of our meta-analysis suggest that low skeletal muscle mass is a significant predictor of long-term mortality in AAA patients undergoing surgical repair. Current evidence is limited by retrospective nature of data and variability in defining and measuring low skeletal muscle mass. There is a need for future prospective studies defining the optimal cut-off of low skeletal muscle mass in different populations.

### Authors’ contributions:

**JC** conceived and designed the study.

**YX, YL and HZ** collected the data and performed the analysis.

**JC** was involved in the writing of the manuscript and is responsible for the integrity of the study.

All authors have read and approved the final manuscript.
